# Solid-State Polymerization of Poly(Ethylene Furanoate) Biobased Polyester, II: An Efficient and Facile Method to Synthesize High Molecular Weight Polyester Appropriate for Food Packaging Applications

**DOI:** 10.3390/polym10050471

**Published:** 2018-04-25

**Authors:** Nejib Kasmi, George Z. Papageorgiou, Dimitris S. Achilias, Dimitrios N. Bikiaris

**Affiliations:** 1 Laboratory of Polymer Chemistry and Technology, Department of Chemistry, Aristotle University of Thessaloniki, GR-541 24 Thessaloniki, Macedonia, Greece; nejibkasmi@gmail.com (N.K.); axilias@chem.auth.gr (D.S.A.); 2 Chemistry Department, University of Ioannina, P.O. Box 1186, 45110 Ioannina, Greece

**Keywords:** poly(ethylene furanoate), solid-state polymerization, high molecular weight, thermal properties, polyester, remelting process

## Abstract

The goal of this study was to synthesize, through a facile strategy, high molecular weight poly(ethylene furanoate) (PEF), which could be applicable in food packaging applications. The efficient method to generate PEF with high molecular weight consists of carrying out a first solid-state polycondensation under vacuum for 6 h reaction time at 205 °C for the resulting polymer from two-step melt polycondensation process, which is catalyzed by tetrabutyl titanate (TBT). A remelting step was thereafter applied for 15 min at 250 °C for the obtained polyester. Thus, the PEF sample was ground into powder, and was then crystallized for 6 h at 170 °C. This polyester is then submitted to a second solid-state polycondensation (SSP) carried out at different reaction times (1, 2, 3.5, and 5 h) and temperatures 190, 200, and 205 °C, under vacuum. Ultimately, a significant increase in intrinsic viscosity is observed with only 5 h reaction time at 205 °C during the second SSP being needed to obtain very high molecular weight PEF polymer greater than 1 dL/g, which sufficient for manufacturing purposes. Intrinsic viscosity (IV), carboxyl end-group content (–COOH), and thermal properties, via differential scanning calorimetry (DSC), were measured for all resultant polyesters. Thanks to the post-polymerization process, DSC results showed that the melting temperatures of the prepared PEF samples were steadily enhanced in an obvious way as a function of reaction time and temperature increase. It was revealed, as was expected for all SSP samples, that the intrinsic viscosity and the average molecular weight of PEF polyester increased with increasing SSP time and temperature, whereas the number of carboxyl end-group concentration was decreased. A simple kinetic model was also developed and used to predict the time evolution of polyesters IV, as well as the carboxyl and hydroxyl end-groups of PEF during the SSP.

## 1. Introduction

The search for sustainable biobased alternatives for polymer production has dramatically intensified in recent years, due to an increasing awareness of finite fossil fuel resources and the disrupting climatic effects of greenhouse gas emissions [1,2,3]. For this reason, the interest in biomass has rapidly emerged as a renewable source of chemicals and mainly monomers for bio-based polymers production [4,5]. The demand for this attractive feedstock in nature is motivating both industrial and scientific communities to innovate a new generation of polymers, and therefore, an opening of the way to an all-inclusive sustainability [6]. Extensive research has escalated in recent years into biorefineries technology development, which has shown a burgeoning surge for producing green monomers [7]. 2,5-Furandicarboxylic acid (FDCA) is the most promising rigid bio-based building block, which has been recognized as one of the twelve most important renewable-based monomers [8]. This furan derivative, which may provide a suitable alternative for terephthalic acid, can be prepared by catalytic oxidation of 5-hydroxymethylfurfural (HMF) derived from C6 sugars or polysaccharides [9,10]. In fact, extensive efforts made up to date on the synthesis of different homopolyesters derived from the renewable-based monomer (FDCA) and various diols [11,12,13,14,15,16,17,18,19]. The most successful furanic biobased polyester is poly(ethylene furan dicarboxylate) (PEF), which is produced from 2,5-furandicarboxylic acid (2,5-FDCA) and ethylene glycol. It is a fully biosourced alternative to its commercial analogue polyethylene terephthalate (PET), produced from petroleum-derived terephthalic acid [20]. Extensive research efforts were triggered since the last decade towards PEF, and its historical progress has been extensively described in two recent extended reports [21,22].

Recently, intensive investigations have been conducted on the commercial polyester (PEF) and its interest is increasing in a spectacular way, due to its renewable nature and promising features. Compared to PET, PEF showed excellent thermal properties, i.e., its processability at lower temperatures due to a lower melting temperature (*T*_m_), and the ability to withstand high temperatures thanks to a higher glass transition temperature (*T*_g_) [23], as well as it is characterized by greatly improved thermal stability up to 320 °C [24]. An impressive 10–27-fold and 19-fold reduction has been reported in oxygen and carbon dioxide permeability, respectively, for PEF compared to PET [25]. Other attractive properties, such as excellent mechanical properties [26], reduced carbon footprint [27], and ability to formulate in films, fibers, and mostly bottles make PEF an appealing substituent to PET [28]. The combination of all PEF features aforementioned are suitable for use as bottles in food and beverage packaging. In 2010, manufacturing of PEF using Avantium’s YXY technology has been started by Avantium in Netherlands [29] for typical applications for packaging of water and fibers, alcoholic beverages, and soft drinks, among others.

Apart from several reports dealing with the emerging topic of PEF by highlighting on its attractive features compared with its analog PET, a drawback still currently an obstacle of interest for researchers, besides the undesired yellowing of the final polyester, as well as its high brittleness, is the production of high molecular weight PEF, which ensures, therefore, the resulting polymer processing in a large safety without any deterioration of its mechanical properties. The decomposition of 2,5-FDCA at high reaction temperatures during melt polycondensation reactions, as well as the important role in the molecular weight increase of the catalyst type used could be the main cause for the emergence of the low molecular weight defect for PEF.

Solid-state polymerization (SSP), performed under mild conditions, has numerous potential advantages and a strong industrial interest to overcome the polymers’ molecular weight limitations obtained from the melt methods [30]. This well-known technique is extensively employed in industry as an extension of the melt polycondensation to produce high molecular weight polyesters with improved properties suitable for wide range of applications (e.g., bottles, films, and fiber production). This competitive process to conventional melt polycondensation, involving heating of the starting partially crystalline polyester at a temperature between its glass transition temperature (*T*_g_) and its melting point (*T*_m_), is used mainly for PET manufacturing to get over its relatively low molecular weight [31,32,33,34,35,36,37,38].

PEF has been the topic of a significant number of publications addressed on its biaxial orientation [39], thermal properties, glass transition, mechanical properties, and isothermal or non- isothermal crystallization [40,41,42,43,44,45,46,47,48,49], as well as investigations on the synthesis and full characterization of this biobased polyester have been well discussed [50,51,52,53,54,55,56,57,58,59].

Surprisingly, although there are numerous studies on PEF synthesis and characterization, only very few publications deal with the industrially relevant process (SSP) of PEF, which leads to manufacturing of high-molecular weight polyester. To date, only three preceding reports [60,61] have been addressed on SSP of PEF as a third stage after two-step melt polycondensation process.

In this context, Knoop et al. [62] have managed to apply SSP method under reduced pressure for PEF, using Ti(IV)-isopropoxide as catalyst. This study, which aims to increase the polymer degree of polymerization during several hours, revealed that PEF with molecular weight of 25.000 g·mol^−^^1^ was obtained after 24 h SSP, and it was increased to 83.000 g·mol^−^^1^ after 72 h SSP of heating at 180 °C. The goal of this report was chiefly focused on the crystallization investigation, and its influence on the mechanical properties of high molecular weight polyester PEF. As it was presented by Hong [63], SSP has been recently carried out for PEF. It was found that the IV increased from 0.6 to 0.64 and 0.72 dL/g after 24 and 48 h of SSP reaction time, respectively.

The current report extends the very limited studies, available nowadays in literature, regarding the synthesis via SSP of high molecular weight PEF. Such work is necessary to enable large-scale industrial applications in PEF-market. In the present study, a facile and efficient method, which is never applied to PEF, has been revealed to effectively circumvent the limited molecular weight of this biobased polyester, and thereafter, the access to very high intrinsic viscosity (IV), up to 1.02 dL/g, appropriate to several applications, such as frozen food trays, tire-cord applications, and principally, for bottle manufacturing [64,65,66,67].

The efficiency of the developed method herein was compared, regarding molecular weight increase, with resulting PEF samples from the application of one SSP reaction to two prepolymers having different initial IV values.

The effect of the temperature and reaction time on the molecular weight increase of the obtained polyester PEF was studied in detail using both experimental measurements and a simple kinetic theoretical model.

## 2. Experimental

### 2.1. Materials

2,5-Furan dicarboxylic acid (2,5-FDCA, purum 97%), ethylene glycol (99.8%), and tetrabutyl titanate (TBT) (97%) catalyst was purchased from Aldrich Co. (Chemie GmbH, Unna, Germany). All other materials and solvents used were of analytical grade.

### 2.2. Synthesis of 2,5-Dimethylfuran-Dicarboxylate(DMFD)

DMFD was prepared as described in the reported procedure [49], whereby a reaction was performed into a round bottom flask (500 mL) in presence of 2,5-furandicarboxylic acid (15.6 g), 200 mL of anhydrous methanol, and 2 mL of concentrated sulfuric acid. The mixture was refluxed for 5 h. The excess of methanol was removed by distillation and filtration was performed out through a disposable Teflon membrane filter (Chemie GmbH, Unna, Germany). During filtration, DMFD was precipitated as white powder, and then 100 mL of distilled water was added, after cooling. Na_2_CO_3_ 5% *w*/*v* was added during stirring while pH was measured continuously to neutralize, partially, the dispersion. DMFD was recuperated as white powder, which was collected by filtration and washed with distilled water, and after drying, was recrystallized with a mixture of 50/50 *v*/*v* methanol/water. According to this procedure, white needles of DMFD were prepared (yield about 83%) with melting point 115 °C, and purity measured by hydrogen nuclear magnetic resonance (^1^H NMR) 99.5% (Bruker spectrometer, Bremen, Germany).

### 2.3. Polyester Synthesis

The PEF polyesters were prepared through the two-stage melt polycondensation method (esterification and polycondensation) in a glass batch reactor as described in our previous work [13]. DMFD and ethylene glycol at a molar ratio of diester/diol = 1:2 were charged with 400 ppm of TBT as catalyst into the reaction tube of the polyesterification apparatus. The reaction mixture was heated under controlled argon flow for 2 h at a temperature of 160 °C, for an additional 1 h at 170 °C, and finally, at 180–190 °C for 1 h. The transesterification stage (first step) was considered complete after the collection of almost all the theoretical amount of methanol, which was removed from the reaction mixture by distillation and collected in a graduated cylinder. In the second stage of polycondensation, the vacuum was gradually reduced to 5.0 Pa over a time of about 30 min, to remove the excess diol, to avoid excessive foaming, and furthermore, to minimize oligomer sublimation, which is a potential problem during melt polycondensation. The temperature was gradually increased, during this time interval, to 230 °C, while stirring speed was also increased to 720 rpm. The reaction was maintained for 2 h at this temperature. After the polycondensation reaction was completed, PEF sample was removed from the reactor, milled, and washed with methanol.

### 2.4. Solid-State Polycondensation

Solid-state polymerization (SSP) was performed using an apparatus involving five volumetric flasks (100 mL) which were connected to a vacuum line, and were immersed in a potassium nitrate/sodium nitrite thermostated bath, having a precision within ±0.5 °C. Crystallized PEF (2 g) with a particle size fraction of −0.40 + 0.16 mm was charged in each one of the volumetric flasks under vacuum, stabilized beneath 3 and 4 Pa. The reaction temperature was kept constant at 190, 200, or 205 °C. The reaction flasks were withdrawn from the bath after 1, 2, 3.5, and 5 h for analysis of the PEF sample’s intrinsic viscosity (IV), to identify the molecular weight of the resulting PEF samples, as well as measuring the carboxyl end-group concentration (COOH). The protocol mentioned above was conducted for two prepolymer PEF samples obtained from melt polycondensation procedure with different initial IV values (0.28 and 0.38 dL/g). The resulting PEF polyesters from SSP are respectively named PEF /TBT.1 and PEF/TBT.2.

The effective method used herein to increase the molecular weight of PEF involves the application of one SSP for 6 h at 205 °C, followed by a remelting step of the obtained polyester for 15 min at 250 °C, and thereafter, a second SSP reaction was applied at different reaction times (1, 2, 3.5, and 5 h) and temperatures 190, 200, and 205 °C, under vacuum. The resulting PEF polyesters are labeled as PEF/TBT.3.

### 2.5. Polyester Characterization

#### 2.5.1. Intrinsic Viscosity Measurement

For intrinsic viscosity [η] measurements, PEF samples (1 wt %) have been dissolved in a mixture of phenol/tetrachloroethane (60:40 *w*/*w*) at 90 °C, and their flow time was measured using an Ubbelohde viscometer (Schott Gerate GMBH, Hofheim, Germany) at 25 °C. The [η] value of each sample was calculated using the following Solomon-Ciuta equation:

[η] = [2{*t*/*t*_0_ − ln(*t*/*t*_0_) – 1}]^1/2^/*c* (1)

where *c* is the concentration of the solution; *t*_0_ the flow time of pure solvent; and *t*, the flow time of solution. Three different measurements were repeated for each sample to ensure the accuracy of the results, and the average value was calculated.

#### 2.5.2. Molecular Weight

The number average molecular weight (${\bar{M}}_{n}$) of the PEF polyester samples was calculated from the intrinsic viscosity [η] values, using the Berkowitz equation [68], as was modified in our previous work [69]:

(2) $${\bar{M}}_{n}=3.29\times 10^{4}{\left[\eta \right]}^{1.54}$$

#### 2.5.3. End-Group Analysis

Carboxyl end-group content (C.C.) of the PEF polyesters was determined according to Pohl’s method, by titrating a solution of the polyester in benzyl alcohol/chloroform mixture. NaOH solution in benzyl alcohol was used as standard solution, and phenol red as indicator [70]. Three different measurements were performed for each sample, and the average value was calculated.

#### 2.5.4. Differential Scanning Calorimetry (DSC)

Differential scanning calorimetry (DSC) study of PEF was carried out on a Perkin-Elmer, Pyris Diamond DSC differential scanning calorimeter (Perkin-Elmer, Waltham, MA, USA), calibrated with high purity indium and zinc standards. For each measurement, a sample of 7 ± 0.1 mg was sealed in aluminum pans, and was then scanned in the instrument from 30 to 240 °C at a heating rate of 20 °C/min under nitrogen flow (50 mL/min). The melting temperature (*T*_m_), the heat of fusion (Δ*H*_m_), and the glass transition temperature (*T*_g_) the of the PEF samples were determined from these scans.

## 3. Modeling the PEF SSP Kinetics

### 3.1. Reaction Mechanism

The reactions taking place during SSP of PEF include polycondensation/transesterification, esterification, thermal degradation, and side reactions of vinyl end-groups [31], and they are illustrated in the following Equations (3)–(6). In these equations, *k*_1_, *K*_1_ and *k*_2_, *K*_2_ denote the forward and equilibrium rate constants of transesterification and esterification reactions, respectively, *k*_d_ and *k*_v_ refer to the kinetic rate constants of the degradation and polycondensation of vinyl end-group reactions, which are considered one way.

Polycondensation/transesterification 
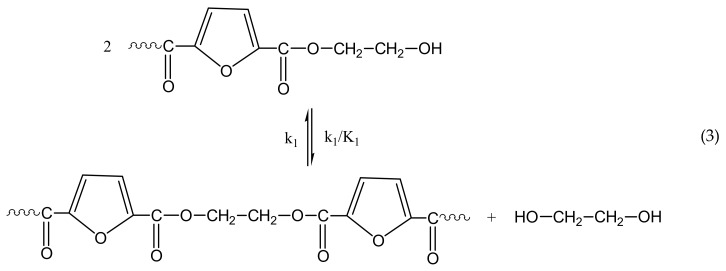


Esterification 
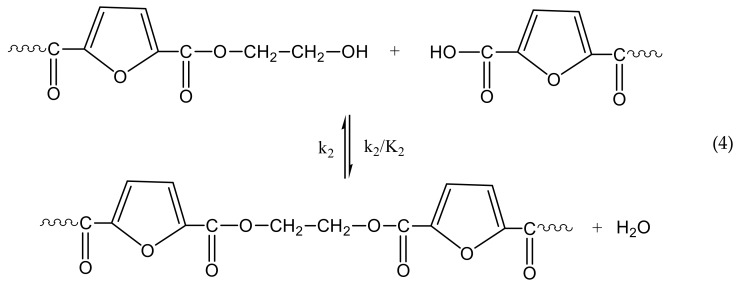


Thermal degradation 
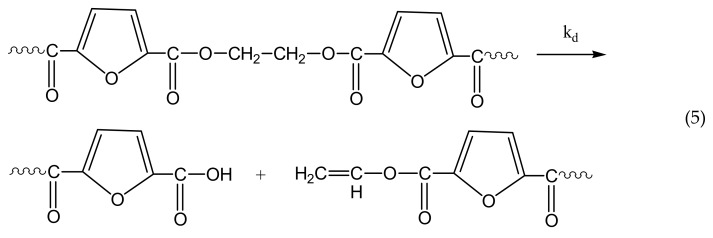


Polycondensation of vinyl end-groups 
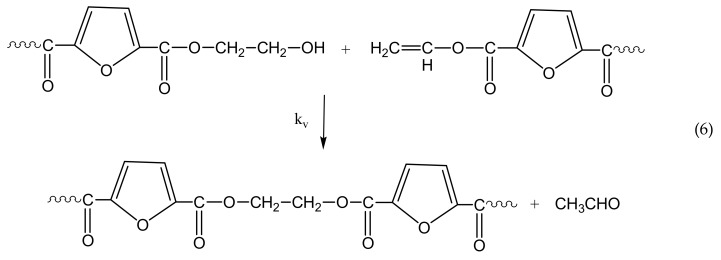


The molecular weight of the polymer is increased by two reactions: in the first, Equation (3), two hydroxyl end-groups react, and ethylene glycol is produced. In the second, Equation (4), a carboxyl end-group reacts with a hydroxyl, and water is released as byproduct. By contrast, when thermal degradation takes place (Equation (5)), the molecular weight of the polyester can be decreased from the cleavage of an ester bond in the macromolecular chain, generating a vinyl ester end-group and a carboxyl end-group. In addition, acetaldehyde may be released from the side reaction of a vinyl ester end-group with a hydroxyl end-group, resulting also in an increase of the molecular weight (Equation (6)). The overall reaction rate is influenced by a combination of several factors, including intrinsic reaction kinetics, change of polymer degree of crystallization, and diffusional limitations of the reactive end-groups, and of the desorbing volatile byproducts (i.e., glycol and water) [71].

### 3.2. Simplified Mathematical Model

The problem of modelling the SSP kinetics is complicated, since, besides chemical kinetics, describing the rate of change of the concentration of the species as a function of time, diffusion phenomena should be incorporated, which results in additional variation with the distance from the interface [31]. Thus, two independent variables are introduced, resulting in a set of partial differential equations that should be solved, including several kinetic, diffusional, and crystallization parameters [72]. Using such complicated models to simulate a few experimental data points is out of any physical meaning. Since in this investigation, only five data points were measured at each experimental condition, a simple kinetic model was adopted afterAgarwal and co-workers [73,74]. This approach was originally developed for the solid-state polycondensation of PET, and successfully applied by our group in modelling the SSP of PET with several nanoadditives, as well as of PEF with nanoadditives [75,76,77].

In order to develop the mathematical model, several assumptions were made, including the following:

- All kinetic rate constants are considered independent of polymer chain length (only end-group reactivity is considered).
- Backward reactions in Equations (3) and (4) are eliminated, due to the fast removal of the water and ethylene glycol, produced in the reaction mixture, caused by the application of high vacuum (beneath 3 and 4 Pa).
- Due to the performance of the polycondensation at relatively low temperatures (i.e., 190–205 °C), no side reactions for the formation of acetaldehyde or thermal degradation are considered (Equations (5) and (6) are eliminated).
- Diffusional limitations on account of desorbing volatile species are neglected.
- Then, the rate of change of hydroxyl [OH] and carboxyl [COOH] end-groups can be described by the following expressions [73,74]:
  (7) $$\frac{d{\left[\mathrm{OH}\right]}_{t}}{dt}=-2k_{1}{\left[\mathrm{OH}\right]}_{t}^{2}-{\left[\mathrm{COOH}\right]}_{t}{\left[\mathrm{OH}\right]}_{t}$$
  (8) $$\frac{d{\left[\mathrm{COOH}\right]}_{t}}{dt}=-k_{2}{\left[\mathrm{COOH}\right]}_{t}{\left[\mathrm{OH}\right]}_{t}$$
  where [OH]_t_ and [COOH]_t_ denote the actual “true” hydroxyl and carboxyl end-group concentrations, respectively.

The term “actual hydroxyl and carboxyl end-groups” was introduced by Ma and Agarwal [73,74], in order to account for the slowdown in SSP kinetics at high [η] values. Accordingly, a part of the carboxyl ([COOH]) and hydroxyl end-groups ([OH]) were considered to be rendered temporarily inactive (denoted as [COOH]_i_ and [OH]_i_) and the actual concentration of OH and COOH in Equations (7) and (8) can be expressed as

[OH]_t_=[OH]–[OH]_i_ (9)

[COOH]_t_=[COOH]–[COOH]_i_ (10)

where [OH], [COOH] and [OH]_i_, [COOH]_i_ denote the concentration of the total and temporarily inactivated OH and COOH end-groups, respectively.

Moreover, the number average molecular weight is expressed as

(11) $${\bar{M}}_{n}=\frac{2}{\left[\mathrm{COOH}\right]+\left[\mathrm{OH}\right]}$$

Equations (7) and (8), together with Equations (2) and (9)–(11), constitute a set of ordinary differential equations which can be easily solved numerically using a varying step-size Runge-Kutta method, to give results on the variation of the intrinsic viscosity and the concentration of –COOH and –OH end-groups as a function of time. Four adjustable parameters, namely *k*_1_, *k*_2_, [OH]_i_, and [COOH]_i_, are estimated at each experimental condition by simultaneous fitting of the values of [OH], [COOH], and IV to the experimental data points as a function of time.

## 4. Results and Discussion

**Figure 1:**
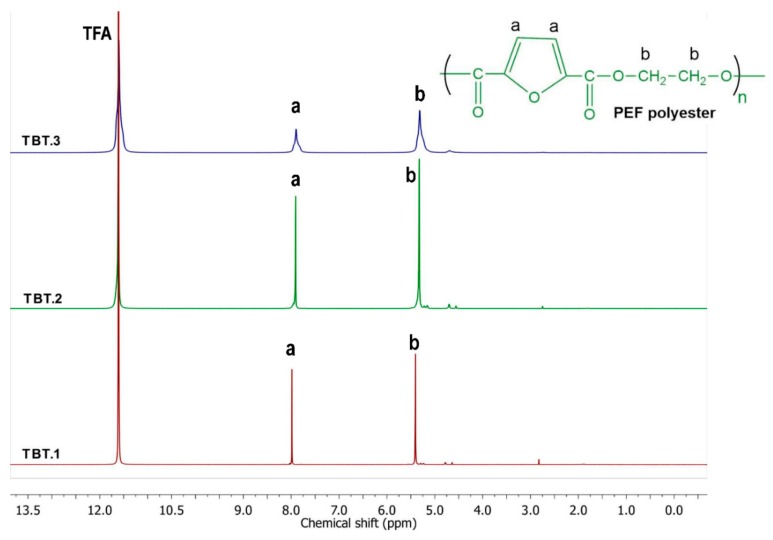
^1^H NMR spectra of poly(ethylene furanoate) (PEF)/ tetrabutyl titanate (TBT) samples.

The chemical structure of the initial polyesters before carrying out the SSP procedure (PEF/TBT.1, PEF/TBT.2, and PEF/TBT.3) was confirmed by ^1^H NMR spectroscopy, as shown in Figure 1. The resonances appeared (b) at 5.4 for PEF/TBT.1, 5.32 for PEF/TBT.2, and 5.31 ppm for PEF/TBT.3 correspond to the methylene protons of the ethylene group. The peaks labelled (a) at 7.98, 7.91, and 7.90 ppm were respectively assigned to the ring protons (2 H, s) of PEF/TBT.1, PEF/TBT.2, and PEF/TBT.3.

### 4.1. Kinetic Study of the Solid-State Polymerization of PEF

**Figure 2:**
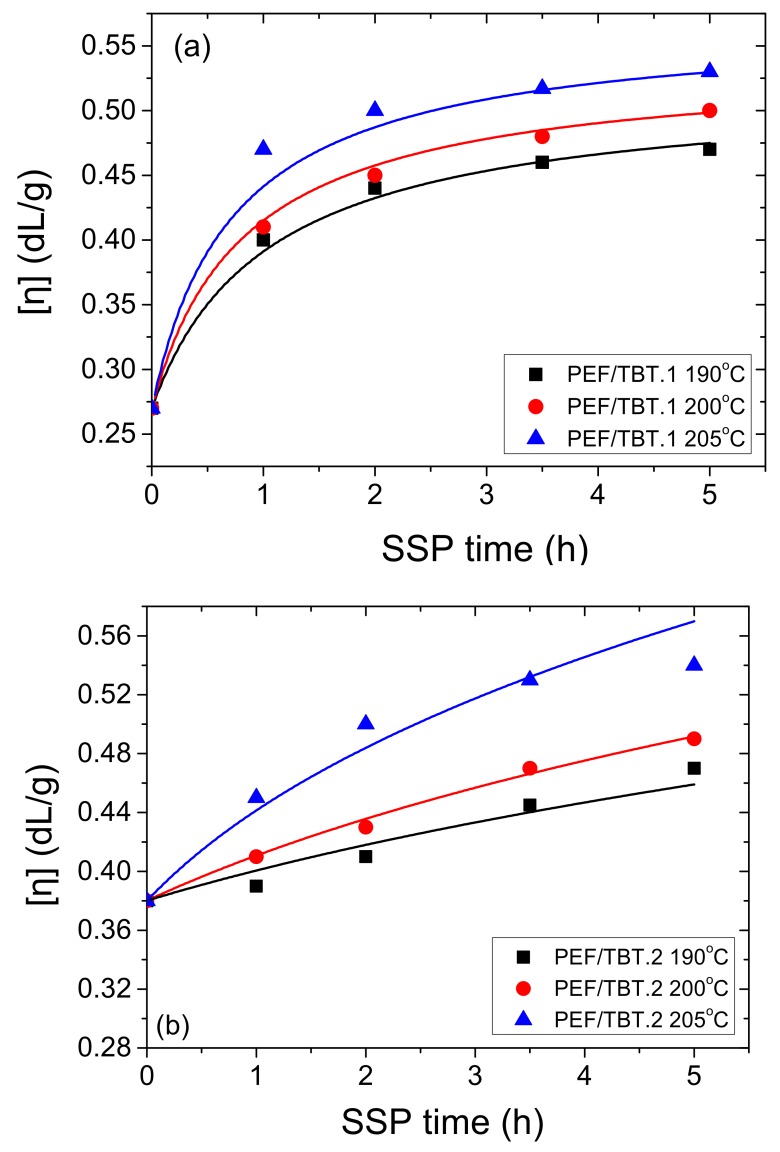

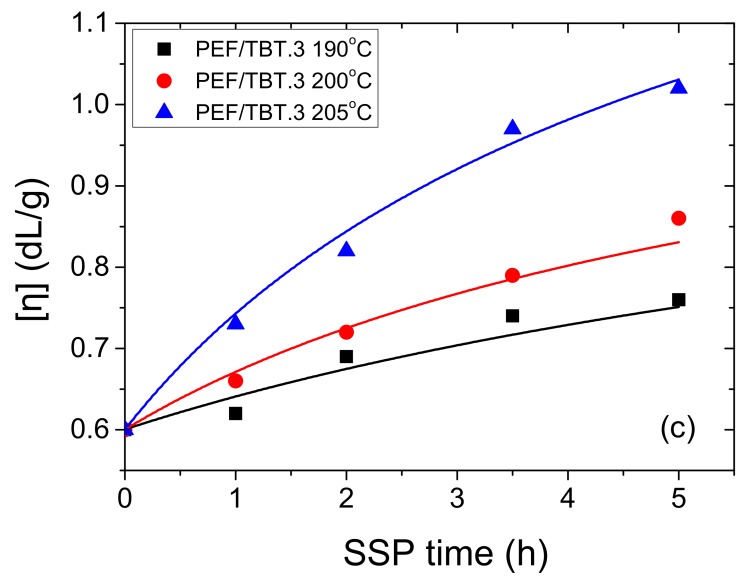
Variation of the intrinsic viscosity with time during solid-state polymerization (SSP) of PEF using TBT catalyst and three different initial IV values; PEF/TBT.1 (**a**), PEF/TBT.2 (**b**), and PEF/TBT.3 (**c**) at different temperatures. Continuous lines represent the theoretical kinetic model simulation results.

As it was also pointed in part I of this research [58], SSP of PEF results in polyesters having increased average molecular weights. The effect of temperature and time on the molecular weight increase during SSP of PEF with TBT catalyst was also investigated here. Two initial PEF samples were employed, having initial IV equal to 0.27 and 0.38 dL/g, and given the names PEF/TBT.1 and PEF/TBT.2, respectively. These IV values correspond to polyesters having initial average degree of polymerization equal to 24 and 40, respectively. As it can be seen in Figure 2, the IV of neat PEF starting at 0.27 dL/g, increases to 0.47, 0.50, and 0.53 dL/g after 5 h of SSP at 190, 200, and 205 °C, respectively. Similar final IV values (i.e., 0.47, 0.50, and 0.54 dL/g after 5 h of SSP at 190, 200, and 205 °C) were measured for the polyester starting at 0.38 dL/g. Increased temperatures favor the increase in the IV values, since both esterification and transesterification reactions are accelerated. Furthermore, diffusion of byproducts produced (such as water and ethylene glycol) is much slower at low SSP temperatures. For these reasons, by increasing the SSP temperature to 205 °C, the IV increase is much higher compared to that at 190 °C (Figure 2a,b). At temperatures close to the melting point of PEF, the macromolecular chains have higher mobility and thus, hydroxyl end-groups react with carboxyl end-groups more easily, joining the macromolecular chains and increasing the molecular weight of PEF. Crystallinity plays also an important role since as higher it is as lower will be the diffusion rate of formed byproducts.

PEF samples, described above, at the end of the SSP, show a relatively low intrinsic viscosity, ranging from 0.47 to 0.54 dL/g. These low values also correspond to low molecular weights, resulting in polyesters having inferior mechanical performance, which is not appropriate for several promising applications. One way to increase the average molecular weight of a polymer conducted after SSP is to employ the so-called remelting process. This is widely applied in the industrial production of polyamides [78,79,80,81], and to a lesser extent, in PET [82,83]. Although many papers have been reported on the advantageous features of the remelting process with respect to the MW increase, to the best of our knowledge, there are no such studies in applying this technique to PEF. In this context, this is the first time that synthesis of PEF is undertaken via this method, in order to achieve a high molecular weight, and consequently, to overcome the inferior properties of the resulting polyester. Therefore, this procedure was applied on PEF/TBT sample for 30 min at 240 °C under argon atmosphere, preceded by SSP involving the same PEF polyester (initial sample with IV = 0.38 dL/g) at 205 °C for 6 h. Once the reaction was over, the polymer was cooled at room temperature, and then, a second SSP reaction was conducted at time intervals 1, 2, 3.5, and 5 h at constant temperatures 190, 200, and 205 °C under vacuum. The evolution of the intrinsic value of the resulting PEF/TBT.3 polyester is depicted in Figure 2c. The IV of PEF starts at 0.61 dL/g, and increases to 0.76, 0.86, and 1.02 dL/g after 5 h of SSP at 190, 200, and 205 °C, respectively. As can be seen, a higher increase of the polymerization rate has been proved, especially at SSP temperatures of 205 °C. This effect is due to the remelting features, which begets a redistribution/homogenization of the reactive end-group separation, and on the other hand, it reduces the polyester water content, which occurs during remelting, and afterwards, making end-group diffusion much easier [30,81].

The calculated number average molecular weight, *M*_n_, as well as the corresponding number-average degree of polymerization from the experimentally measured IV of all prepared samples are summarized in Table 1. The final PEF polyesters obtained after 2, 3.5, and 5 h of SSP at 205 °C for the PEF/TBT.3 sample are characterized by high IV values of 0.82, 0.97, and 1.02 dL/g, corresponding to number average molecular weights of 24,236; 31,392; and 33,920 g·mol^−1^, respectively. These values are much higher compared to the corresponding values obtained for the PEF/TBT.1 and PEF/TBT.2 samples, which are similar at 205 °C, and range from 11,300 to 12,700 g·mol^−1^. It can be stated that the reached IV range is enough to meet specific end-use requirements. It is suitable for packaging applications, such as carbonated beverage bottles and manufacturing of sheet grades for thermoforming [66].

**Figure 3:**
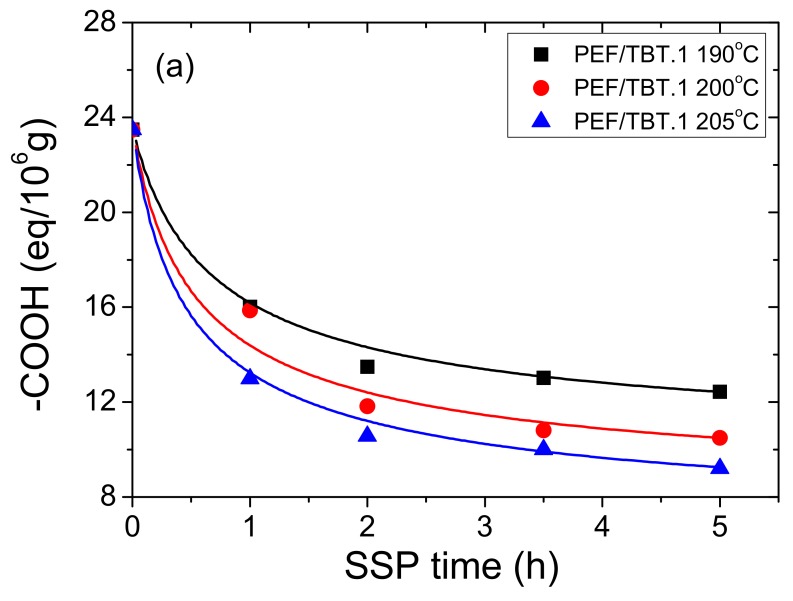

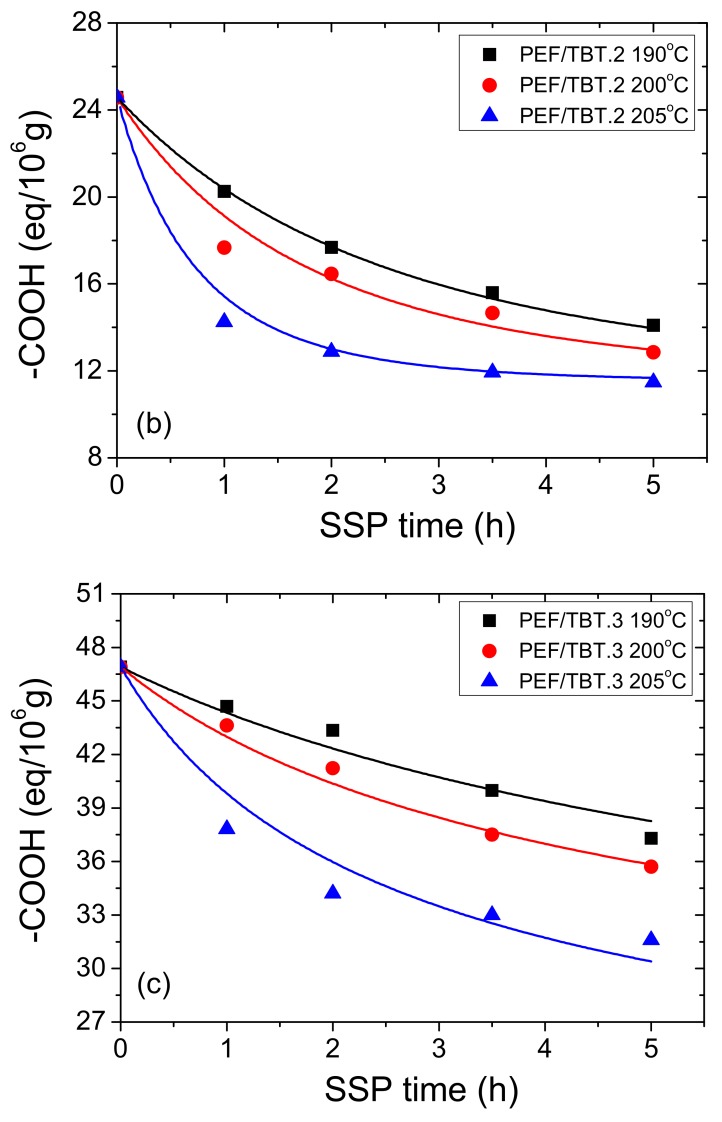
Variation of carboxyl end-groups with time during PEF/TBT.1 (**a**); PEF/TBT.2 (**b**); and PEF/TBT.3 (**c**) SSP at different temperatures. Continuous lines represent the theoretical kinetic model simulation results.

**Figure 4:**
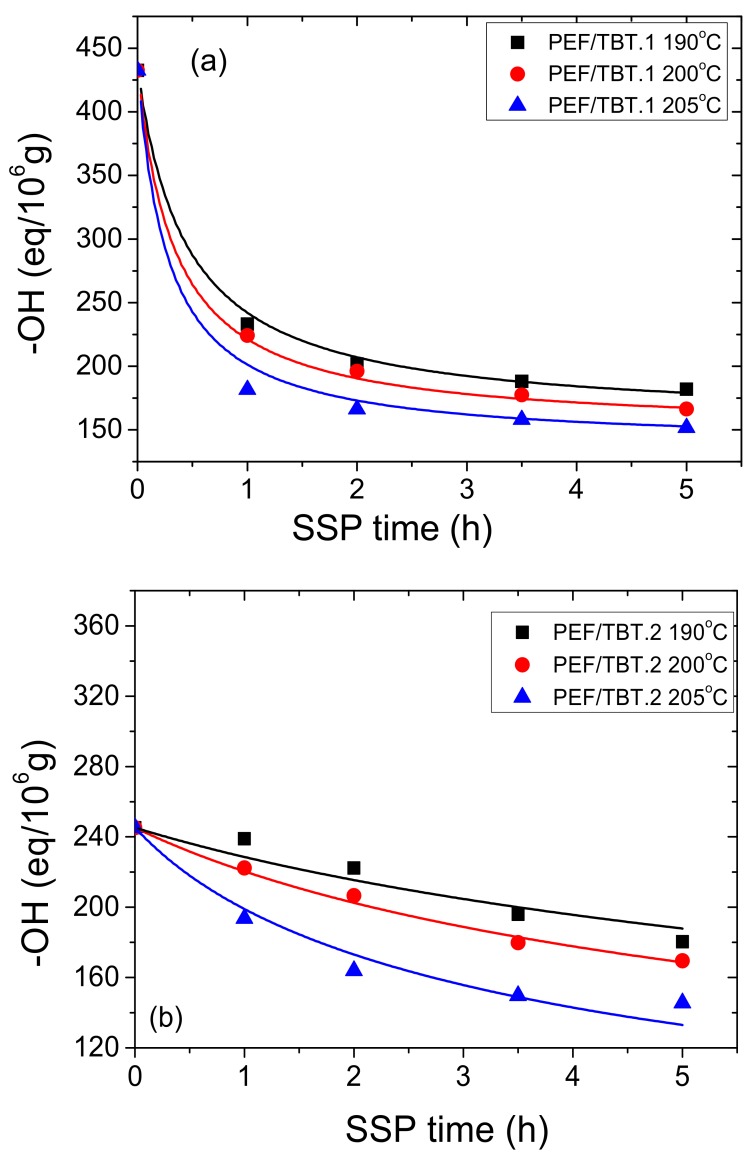

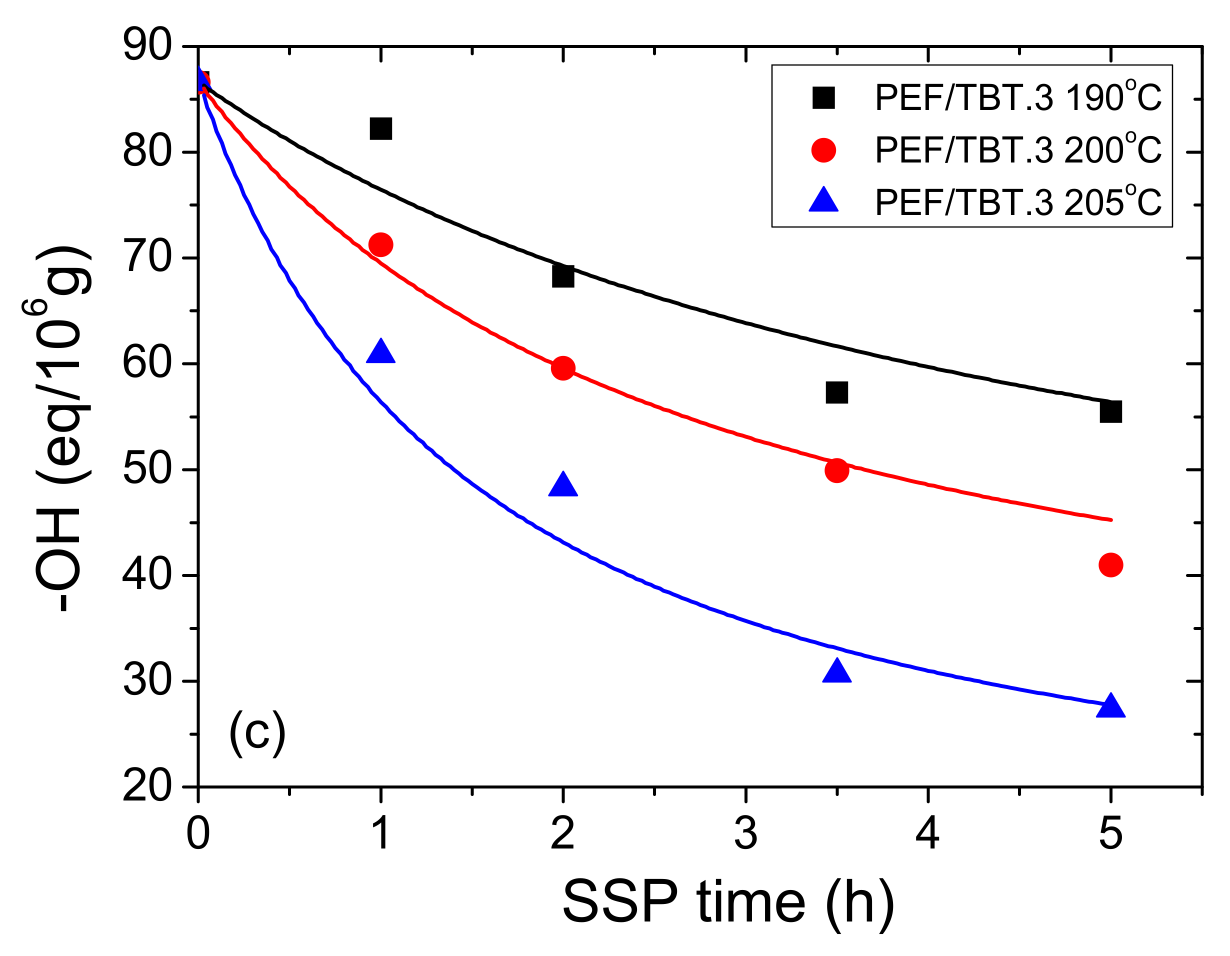
Variation of hydroxyl end-groups with time during PEF/TBT.1 (**a**), PEF/TBT.2 (**b**), and PEF/ TBT.3 (**c**) SSP at different temperatures. Continuous lines represent the theoretical kinetic model simulation results.

Moreover, end-group analysis (–COOH and –OH) was performed on all prepared samples. Figure 3 and Figure 4 show the effect of SSP time and temperature on the –COOH and –OH concentrations of all PEF/TBT samples. It is obvious that the carboxyl and hydroxyl contents decrease with increasing SSP time and temperature, wherein at low SSP temperature (190 and 200 °C), carboxyl end-groups content decreases almost linearly with the SSP time, while it begins to sharply deviate from the linear relationship at 205 °C. Carboxyl end-groups start at approximately 24 eq/10^6^ for both PEF/TBT.1 and PEF/TBT.2, and after 5 h of SSP, depending on the temperature, reach values ranging from 9 to 12 eq/10^6^ and 11 to 14 eq/10^6^ for PEF/TBT.1 and PEF/TBT.2, respectively. Moreover, it was expected that PEF/TBT.3 samples should exhibit a content of carboxyl end-groups much lower compared to those of other PEF/TBT samples, due to their much higher molecular weights. However, this was not the case, as a high COOH concentration of 46.9 eq/10^6^ was obtained after remelting. After 5 h of SSP, the carboxyl content was reduced to 32–37 eq/10^6^ depending on temperature. The main reason for these high values could be the existence of degradation reactions, which take place principally at high temperatures in the melt phase, wherein a high amount of carboxyl end-groups have been easily created. Accordingly, the major degradation reaction responsible for the generation of carboxyl ends happened mainly via the cleavage of an ester bond in the PEF main chain producing vinyl ester and carboxyl end-groups. More impressive results were observed when calculating the hydroxyl content, which in the case of PEF/TBT.1 and PEF/TBT.2, start at high amounts, i.e., 433 and 245 eq/10^6^, respectively, and after 5 h, SSP ranges from 150 to 180 eq/10^6^, depending on temperature. By contrast, in the PEF/TBT.3 samples, the hydroxyl content was very much lower, starting at 87 eq/10^6^, and after 5 h SSP, decreasing to only 55, 41, and 27 eq/106 at 190, 200, and 205 °C, respectively. Thus, it seems, that in this polyester, in contrast to results obtained for PEF/TBT.1 and PEF/TBT.2, the concentrations of –COOH and –OH are similar.

Furthermore, in order to provide results on the kinetic rate constants of the esterification and polycondensation reactions, the theoretical kinetic model presented in Section 3, was employed. Differential Equations (7) and (8) were solved numerically together with Equations (2), (9)–(11), and IV values, as well as the concentration of hydroxyl and carboxyl end-groups were obtained as a function of SSP time. The best-fit values of the parameters *k*_1_, *k*_2_, [OH]_i_, and [COOH]_i_ were estimated using the experimental data presented in Figure 2, Figure 3 and Figure 4 for all PEF/TBT samples at all temperatures. Optimized values are illustrated in Table 2. Results of the theoretical simulation curves are included as continuous lines in the abovementioned figures. As it can be seen, theoretical simulation curves follow satisfactorily the experimental data at all different temperatures. Slight discrepancies could be attributed to the assumptions made, when developing the simple kinetic model. From an inspection of the values reported in Table 2, it seems that the values of the transesterification rate constant, *k*_1_, are lower than that of the esterification rate constant, *k*_2_, for PEF/TBT.1 and PEF/TBT.2, following the concentration of –COOH, which is lower compared to that of –OH for these polyesters. However, in PEF/TBT.3, where both concentrations are similar, the model also resulted in similar *k*_1_ and *k*_2_ values. Decreased *k*_2_ estimated for the PEF/TBT.3 compared to the other polyesters is a direct consequence of the restricted mobility of reactive end-groups (e.g., hydroxyl and carboxyl) to come into close proximity and react, which is facilitated by the polyester’s higher molecular weight.

In addition, from Table 2, it was estimated that the best fit value for the hydroxyl inactive groups, [OH]_i_ (meaning those which are inaccessible to react), are always lower in PEF/TBT.3 compared to the other polyesters, while they are always reduced with increasing temperature. An increase in temperature improves the mobility of the polymer chains, and thus, reduces the number of inactive end species. The lower values in PEF/TBT.3 is a direct consequence of the always lower –OH end-group concentration measured at all reaction times and temperatures compared to other polyesters. Concerning the inactive [COOH]_i_, the values are always low enough for all polyesters.

Finally, both kinetic rate constants were correlated with temperature using an Arrhenius-type expression. As expected, the values of all rate constants increase with SSP temperature, in accordance with the mobility and activity of the chain ends. When plotting ln(*k*) vs 1/T, good straight lines were obtained with a correlation coefficient greater than 0.90. From the slope of these straight lines, the activation energies for the transesterification, *E*_1_, and esterification, *E*_2_, reactions were determined (Table 3). It should be noted that the estimation of the activation energies using only 3 experimental data points (at the three investigated temperatures) results in a somehow great uncertainty in the values denoted by their high standard deviation. Thus, a safe conclusion concerning the activation energies cannot be set.

### 4.2. Thermal Analysis of Solid-State Polymerization PEF Polyester Samples

**Figure 5:**
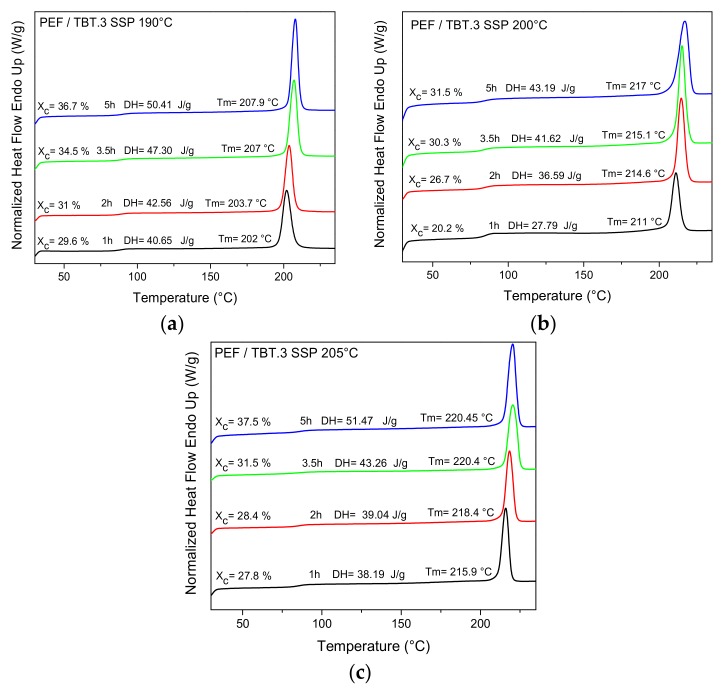
DSC thermograms of PEF/TBT.3 samples prepared after SSP at different temperatures and times (**a**) 190 °C, (**b**) 200 °C, and (**c**) 205 °C.

Differential scanning calorimetric (DSC) measurements showed that SSP strongly affects the thermal properties of the PEF samples. The results of the melting behavior of the PEF samples at different temperatures and reaction times are provided in Figure 5 (as well as in Figures S1 and S2 in the Supporting Information). For all samples, increasing either the SSP time or the SSP temperature shifts the endothermic melting peaks to a higher temperature, with an accompanying increase of the crystallinity. The increased molecular weight of the polyester produced during SSP procedure is responsible for the increase in the melting points, as well as the increase in the sharpness of the melting peaks.

**Figure 6:**
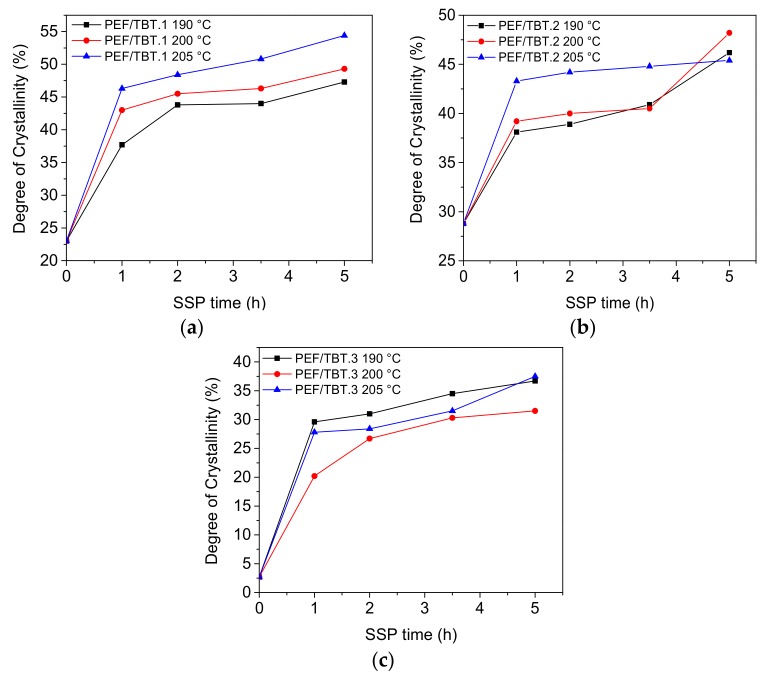
Effect of SSP time and temperature on the evolution of the degree of crystallinity of PEF samples: (**a**) PEF/TBT.1, (**b**) PEF/TBT.2, (**c**) PEF/TBT.3.

Table 4 shows the degree of crystallinity values (*X*_c_) for all SSP PEF samples. The latter were calculated from measured melting enthalpy (Δ*H*_m_) using the heat of fusion value for the pure crystalline PEF polyester found in previous report to be about 137 J·g^−1^ [13]. As illustrated in Figure 6, the degree of crystallinity reached the highest value for PEF/TBT.1, and then showed a slightly lower value for PEF/TBT.2, while the polyester PEF/TBT.3 exhibits much lower *X*_c_. Considering though, the fact that the SSP procedure takes place in the amorphous regions, and as the mobility of the diffusion rate and the polymer chain end-groups, which are concentrated only in the amorphous phase of the semi-crystalline polyester, are affected by the crystallinity, it can be inferred that the rapidly increasing molecular weight rate/IV values of PEF/TBT.3, when compared with the other two polyester samples, is due, in fact, to the lowest degree of crystallinity, as presented in Figure 6. This implies that the scape of low molecular weight byproducts got more facile, thus, the increase of molecular weight became faster. This explanation is in good accordance with the *M*_n_/IV values obtained in this study.

## 5. Conclusions

The present work is, to the best of our knowledge, the first study which investigated the feasibility of SSP after remelting process to synthesize PEF polyesters. Obviously, by introducing a remelting step in the SSP procedure, the latter was found to be a very efficient method that leads to the production of PEF with very high molecular weight appropriate for food packaging applications. The effect of the remelting on the SSP kinetics of PEF was investigated at several temperatures, both experimentally and using a simple kinetic model. As expected, the average molecular weight and the intrinsic viscosity of PEF increased as SSP time and temperature increase. This is because the elimination of formed byproducts during both esterification and transesterification reactions that are occurring during SSP, are diffusion controlled. A simple kinetic model was also developed, and used to predict the time evolution of polyesters’ IV, as well as the hydroxyl and carboxyl content during the SSP of PEF. From both the theoretical simulation results and the experimental measurements, it has been demonstrated that the introduction of remelting process in SSP procedure resulted in similar transesterification and esterification kinetic rate constants, as well as in a higher increase of the polymerization rate, and thus, the obtaining of very high molecular weight PEF.

## Figures and Tables

**Table 1:** Number average molecular weights (${\bar{M}}_{n}$, g·mol^−1^) of PEF polyester using TBT catalyst obtained after SSP at different temperatures and times. The value includes, in parentheses, the corresponding number average degree of polymerization.

Temperature (°C)	SSP time (h)	PEF/TBT.1	PEF/TBT.2	PEF/TBT.3
	0	4400 (24)	7400 (40)	15,000 (81)
190	1	8000 (44)	7700 (42)	15,800 (86)
	2	9300 (51)	8300 (45)	18,600 (101)
	3.5	10,000 (54)	10,000 (54)	20,700 (112)
	5	10,300 (56)	10,600 (58)	21,600 (117)
200	1	8300 (45)	8300 (45)	17,400 (90)
	2	9600 (52)	9000 (49)	19,800 (108)
	3.5	10,600 (58)	10,300 (56)	22,900 (124)
	5	11,300 (62)	11,000 (59)	26,100 (142)
205	1	10,300 (56)	9600 (52)	20,300 (110)
	2	11,300 (62)	11,700 (63)	24,200 (132)
	3.5	11,900 (63)	12,400 (67)	31,400 (171)
	5	12,400 (67)	12,700 (69)	33,900 (184)

**Table 2:** Kinetic rate constants of the transesterification and esterification reaction and concentration of temporarily inactivated OH and COOH end-groups at different polycondensation temperatures of PEF/TBT.1, PEF/TBT.2, and PEF/TBT.3.

Sample	Temperature (°C)	*k*_1_·(kg/meq)·h^−1^	*k*_2_·(kg/meq)·h^−1^	[OH]_i_ (meq/kg)	[COOH]_i_ (meq/kg)
PEF/TBT.1	190	39 × 10^−4^	51 × 10^−4^	157	9.5
	200	50 × 10^−4^	64 × 10^−4^	150	7.5
	205	59 × 10^−4^	71 × 10^−4^	138	6.0
PEF/TBT.2	190	2.0 × 10^−4^	22 × 10^−4^	60	11.5
	200	3.2 × 10^−4^	31 × 10^−4^	58	11.5
	205	6.6 × 10^−4^	72 × 10^−4^	52	11.5
PEF/TBT.3	190	15 × 10^−4^	14 × 10^−4^	31	9.0
	200	25 × 10^−4^	21 × 10^−4^	26	9.0
	205	36 × 10^−4^	37 × 10^−4^	13	9.0

**Table 3:** Activation energies and correlation coefficients of the transesterification and esterification reaction of all PEF/TBT polyesters.

Sample	*E*_1_ (kJ/mol)	*R* ^2^	*E*_2_ (kJ/mol)	*R* ^2^
PEF/TBT.1	50.0 ± 4.0	0.996	40.7 ± 0.6	0.999
PEF/TBT.2	137.4 ± 11.4	0.950	133.1 ± 15.1	0.907
PEF/TBT.3	105.3 ± 9.7	0.979	112.5 ± 24.1	0.895

**Table 4:** Degree of crystallinity values (%) of the different SSP PEF/TBT samples.

SSP temperature (°C)	SSP time (h)	PEF/TBT.1	PEF/TBT.2	PEF/TBT.3
	0	23	28.8	2.7
190	1	37.7	38.1	29.6
	2	43.8	38.9	31
	3.5	44	40.9	34.5
	5	47.3	46.2	36.7
200	1	43	39.2	20.2
	2	45.5	40	26.7
	3.5	46.3	40.5	30.3
	5	49.3	48.2	31.5
205	1	46.3	43.3	27.8
	2	48.4	44.2	28.4
	3.5	50.8	44.8	31.5
	5	54.4	45.4	37.5

## Supplementary Materials

The following are available online at http://www.mdpi.com/2073-4360/10/5/471/s1, Figure S1: DSC thermograms of PEF/TBT.1 samples prepared after SSP at different temperatures and times, Figure S2: DSC thermograms of PEF/TBT.2 samples prepared after SSP at different temperatures and times.

## References

[1] Gandini A. (2011). The irruption of polymers from renewable resources on the scene of macromolecular science and technology. Green Chem..

[2] Meier M.A.R., Metzger J.O., Schubert U.S. (2007). Plant oil renewable resources as green alternatives in polymer science. Chem. Soc. Rev..

[3] Vilela C., Sousa A.F., Fonseca A.C., Serra A.C., Coelho J.F.J., Freire C.S.R., Silvestre A.J.D. (2014). The quest for sustainable polyesters—Insights into the future. Polym. Chem..

[4] Gandini A., Lacerda T.M. (2015). From monomers to polymers from renewable resources: Recent advances. Prog. Polym. Sci..

[5] Delidovich I., Hausoul P.J.C., Deng L., Pfützenreuter R., Rose M., Palkovits R. (2016). Alternative Monomers Based on Lignocellulose and Their Use for Polymer Production. Chem. Rev..

[6] Gandini A., Lacerda T.M., Carvalho A.J.F., Trovatti E. (2016). Progress of Polymers from Renewable Resources: Furans, Vegetable Oils, and Polysaccharides. Chem. Rev..

[7] Pellis A., Herrero Acero E., Gardossi L., Ferrario V., Guebitz G.M. (2016). Renewable building blocks for sustainable polyesters: New biotechnological routes for greener plastics. Polym. Int..

[8] Werpy T., Petersen G. (2004). Top Value Added Chemicals from Biomass, Volume I–Results of Screening for Potential Candidates from Sugars and Synthesis Gas.

[9] Thiyagarajan S., Genuino H.C., van der Waal J.C., De Jong E., Weckhuysen B.M., Van Haveren J., Bruijnincx P.C., Van Es D.S. (2016). A Facile solid-phase route to renewable aromatic chemicals from biobased furanics. Angew. Chem. Int. Ed..

[10] Esposito D., Antonietti M. (2015). Redefining biorefinery: The search for unconventional building blocks for materials. Chem. Soc. Rev..

[11] Vannini M., Marchese P., Celli A., Lorenzetti C. (2015). Fully biobased poly(propylene 2,5-furandicarboxylate) for packaging applications: Excellent barrier properties as a function of crystallinity. Green Chem..

[12] Soares M.J., Dannecker P.-K., Vilela C., Bastos J., Meier M.A.R., Sousa A.F. (2017). Poly(1,20-eicosanediyl 2,5-furandicarboxylate), a biodegradable polyester from renewable resources. Eur. Polym. J..

[13] Terzopoulou Z., Karakatsianopoulou E., Kasmi N., Majdoub M., Papageorgiou G.Z., Bikiaris D.N. (2017). Effect of catalyst type on recyclability and decomposition mechanism of poly(ethylene furanoate) biobased polyester. J. Anal. Appl. Pyrolysis.

[14] Papageorgiou D.G., Guigo N., Tsanaktsis V., Exarhopoulos S., Bikiaris D.N., Sbirrazzuoli N., Papageorgiou G.Z. (2016). Fast crystallization and melting behavior of a long-spaced aliphatic furandicarboxylate bio-based polyester, the poly(dodecylene 2,5-furanoate). Ind. Eng. Chem. Res..

[15] Tsanaktsis V., Terzopoulou Z., Nerantzaki M., Papageorgiou G.Z., Bikiaris D.N. (2016). New poly(pentylene furanoate) and poly(heptylene furanoate) sustainable polyesters from diols with odd methylene groups. Mater. Lett..

[16] Tsanaktsis V., Terzopoulou Z., Exarhopoulos S., Bikiaris D.N., Achilias D.S., Papageorgiou D.G., Papageorgiou G.Z. (2015). Sustainable, eco-friendly polyesters synthesized from renewable resources: Preparation and thermal characteristics of poly(dimethyl-propylene furanoate). Polym. Chem..

[17] Zhu J., Cai J., Xie W., Chen P.-H., Gazzano M., Scandola M., Gross R.A. (2013). Poly(butylene 2,5-furan dicarboxylate), a Biobased Alternative to PBT: Synthesis, Physical Properties, and Crystal Structure. Macromolecules.

[18] Terzopoulou Z., Kasmi N., Tsanaktsis V., Doulakas N., Bikiaris D.N., Achilias D.S., Papageorgiou G.Z. (2017). Synthesis and Characterization of Bio-Based Polyesters: Poly(2-methyl-1,3-propylene-2,5-furanoate), Poly(isosorbide-2,5-furanoate), Poly(1,4-cyclohexane dimethylene-2,5-furanoate). Materials.

[19] Carlos Morales-Huerta J., Martínez De Ilarduya A., Muñoz-Guerra S. (2016). Poly(alkylene 2,5-furandicarboxylate)s (PEF and PBF) by ring opening polymerization. Polymer.

[20] De Jong E., Dam M.A., Sipos L., Gruter G.-J.M. (2012). Furandicarboxylic acid (FDCA), A versatile building block for a very interesting class of polyesters. ACS Symp. Ser..

[21] Sousa A.F., Vilela C., Fonseca A.C., Matos M., Freire C.S.R., Gruter G.-J.M., Coelho J.F.J., Silvestre A.J.D. (2015). Biobased polyesters and other polymers from 2,5-furandicarboxylic acid: A tribute to furan excellence. Polym. Chem..

[22] Papageorgiou G.Z., Papageorgiou D.G., Terzopoulou Z., Bikiaris D.N. (2016). Production of bio-based 2,5-furan dicarboxylate polyesters: Recent progress and critical aspects in their synthesis and thermal properties. Eur. Polym. J..

[23] Weinberger S., Canadell J., Quartinello F., Yeniad B., Arias A., Pellis A., Guebitz G.M. (2017). Enzymatic Degradation of Poly(ethylene 2,5-furanoate) Powders and Amorphous Films. Catalysts.

[24] Thiyagarajan S., Vogelzang W.J.I., Knoop R., Frissen A.E., Van Haveren J., Van Es D.S. (2014). Biobased furandicarboxylic acids (FDCAs): Effects of isomeric substitution on polyester synthesis and properties. Green Chem..

[25] Tsanaktsis V., Papageorgiou D.G., Exarhopoulos S., Bikiaris D.N., Papageorgiou G.Z. (2015). Crystallization and Polymorphism of Poly(ethylene furanoate). Cryst. Growth Des..

[26] Burgess S.K., Leisen J.E., Kraftschik B.E., Mubarak C.R., Kriegel R.M., Koros W.J. (2014). Chain Mobility, Thermal, and Mechanical Properties of Poly(ethylene furanoate) Compared to Poly(ethylene terephthalate). Macromolecules.

[27] Eerhart A.J.J.E., Faaij A.P.C., Patel M.K. (2012). Replacing fossil based PET with biobased PEF; process analysis, energy and GHG balance. Energy Environ. Sci..

[28] Burgess S.K., Kriegel R.M., Koros W.J. (2015). Carbon dioxide sorption and transport in amorphous poly(ethylene furanoate). Macromolecules.

[29] PEF: Game-Changing Plastic. https://www.avantium.com/yxy/products-applications/.

[30] Steinborn-Rogulska I., Rokicki G. (2013). Solid-state polycondensation (SSP) as a method to obtain high molecular weight polymers. Part II. Synthesis of polylactide and polyglycolide via SSP. Polimery.

[31] Vouyiouka S.N., Karakatsani E.K., Papaspyrides C.D. (2005). Solid state polymerization. Prog. Polym. Sci..

[32] Papaspyrides C.D., Vouyiouka S.N. (2009). Solid State Polymerization.

[33] Zhang J., Shen X.-J., Zhang J., Feng L.-F., Wang J.-J. (2013). Experimental and modeling study of the solid state polymerization of poly(ethylene terephthalate) over a wide range of temperatures and particle sizes. J. Appl. Polym. Sci..

[34] Li L.-J., Duan R.-T., Zhang J.-B., Wang X.-L., Chen L., Wang Y.-Z. (2013). Phosphorus-Containing Poly(ethylene terephthalate): Solid-State Polymerization and Its Sequential Distribution. Ind. Eng. Chem. Res..

[35] Gantillon B., Spitz R., McKenna T.F. (2004). The Solid State Postcondensation of PET, 1: A Review of the Physical and Chemical Processes Taking Place in the Solid State. Macromol. Mater. Eng..

[36] Karayannidis G.P., Kokkalas D.E., Bikiaris D.N. (1995). Solid-state polycondensation of poly(ethylene terephthalate) recycled from postconsumer soft-drink bottles. II. J. Appl. Polym. Sci..

[37] Bikiaris D., Karavelidis V., Karayannidis G. (2006). A New Approach to Prepare Poly(ethylene terephthalate)/Silica Nanocomposites with Increased Molecular Weight and Fully Adjustable Branching or Crosslinking by SSP. Macromol. Rapid Commun..

[38] Achilias D.S., Bikiaris D.N., Karavelidis V., Karayannidis G.P. (2008). Effect of silica nanoparticles on solid state polymerization of poly(ethylene terephthalate). Eur. Polym. J..

[39] Van Berkel J.G., Guigo N., Kolstad J.J., Sbirrazzuoli N. (2018). Biaxial Orientation of Poly(ethylene 2,5-furandicarboxylate): An Explorative Study. Macromol. Mater. Eng..

[40] Burgess S.K., Mubarak C.R., Kriegel R.M., Koros W.J. (2015). Physical aging in amorphous poly(ethylene furanoate): Enthalpic recovery, density, and oxygen transport considerations. J. Polym. Sci. Part B.

[41] Guigo N., van Berkel J., de Jong E., Sbirrazzuoli N. (2017). Modelling the non-isothermal crystallization of polymers: Application to poly(ethylene 2,5-furandicarboxylate). Thermochim. Acta.

[42] Codou A., Moncel M., Van Berkel J.G., Guigo N., Sbirrazzuoli N. (2016). Glass transition dynamics and cooperativity length of poly(ethylene 2,5-furandicarboxylate) compared to poly(ethylene terephthalate). Phys. Chem. Chem. Phys..

[43] Burgess S.K., Mikkilineni D.S., Yu D.B., Kim D.J., Mubarak C.R., Kriegel R.M., Koros W.J. (2014). Water sorption in poly(ethylene furanoate) compared to poly(ethylene terephthalate). Part 1: Equilibrium sorption. Polymer.

[44] Van Berkel J.G., Guigo N., Kolstad J.J., Sipos L., Wang B., Dam M.A., Sbirrazzuoli N. (2014). Isothermal Crystallization Kinetics of Poly(Ethylene 2,5-Furandicarboxylate). Macromol. Mater. Eng..

[45] Codou A., Guigo N., Van Berkel J., De Jong E., Sbirrazzuoli N. (2014). Non-isothermal Crystallization Kinetics of Biobased Poly(ethylene 2,5-furandicarboxylate) Synthesized via the Direct Esterification Process. Macromol. Chem. Phys..

[46] Burgess S.K., Mikkilineni D.S., Yu D.B., Kim D.J., Mubarak C.R., Kriegel R.M., Koros W.J. (2014). Water sorption in poly(ethylene furanoate) compared to poly(ethylene terephthalate). Part 2: Kinetic sorption. Polymer.

[47] Stoclet G., Gobius Du Sart G., Yeniad B., De Vos S., Lefebvre J.M. (2015). Isothermal crystallization and structural characterization of poly(ethylene-2,5-furanoate). Polymer.

[48] Maini L., Gigli M., Gazzano M., Lotti N., Bikiaris D.N., Papageorgiou G.Z. (2018). Structural investigation of poly(ethylene furanoate) polymorphs. Polymers.

[49] Papageorgiou G.Z., Tsanaktsis V., Bikiaris D.N. (2014). Synthesis of poly(ethylene furandicarboxylate) polyester using monomers derived from renewable resources: Thermal behavior comparison with PET and PEN. Phys. Chem. Chem. Phys..

[50] Pellis A., Haernvall K., Pichler C.M., Ghazaryan G., Breinbauer R., Guebitz G.M. (2016). Enzymatic hydrolysis of poly(ethylene furanoate). J. Biotechnol..

[51] Weinberger S., Haernvall K., Scaini D., Ghazaryan G., Zumstein M.T., Sander M., Pellis A., Guebitz G.M. (2017). Enzymatic surface hydrolysis of poly(ethylene furanoate) thin films of various crystallinities. Green Chem..

[52] Stoclet G., Lefebvre J.M., Yeniad B., Gobius du Sart G., De Vos S. (2018). On the strain-induced structural evolution of Poly(ethylene-2,5-furanoate) upon uniaxial stretching: An in-situ SAXS-WAXS study. Polymer.

[53] Lotti N., Munari A., Gigli M., Gazzano M., Tsanaktsis V., Bikiaris D.N., Papageorgiou G.Z. (2016). Thermal and structural response of in situ prepared biobased poly(ethylene 2,5-furan dicarboxylate) nanocomposites. Polymer.

[54] Jiang M., Liu Q., Zhang Q., Ye C., Zhou G. (2012). A Series of Furan-Aromatic Polyesters Synthesized via Direct Esterification Method Based on Renewable Resources. J. Polym. Sci. Part A.

[55] Rosenboom J.-G., Roo J.D., Storti G., Morbidelli M. (2017). Diffusion (DOSY) ^1^H NMR as an Alternative Method for Molecular Weight Determination of Poly(ethylene furanoate) (PEF) Polyesters. Macromol. Chem. Phys..

[56] Gomes M., Gandini A., Silvestre A.J.D., Reis B. (2011). Synthesis and Characterization of Poly(2,5-furan dicarboxylate)s Based on a Variety of Diols. J. Polym. Sci. Part A.

[57] Gruter G.-J.M., Sipos L., Dam M.A. (2012). Accelerating Research into Bio-Based FDCA-Polyesters by Using Small Scale Parallel Film Reactors. Comb. Chem. High Throughput Screen..

[58] Kasmi N., Majdoub M., Papageorgiou G.Z., Achilias D.S., Bikiaris D.N. (2017). Solid-State Polymerization of Poly(ethylene furanoate) Biobased Polyester, I: Effect of Catalyst Type on Molecular Weight Increase. Polymers.

[59] Terzopoulou Z., Karakatsianopoulou E., Kasmi N., Tsanaktsis V., Nikolaidis N., Kostoglou M., Papageorgiou G.Z., Lambropoulou D.A., Bikiaris D.A. (2017). Effect of catalyst type on molecular weight increase and coloration of poly(ethylene furanoate) biobased polyester during melt polycondensation. Polym. Chem..

[60] Achilias D.S., Chondroyiannis A., Nerantzaki M., Adam K.-V., Terzopoulou Z., Papageorgiou G.Z., Bikiaris D.N. (2017). Solid State Polymerization of Poly(Ethylene Furanoate) and Its Nanocomposites with SiO_2_ and TiO_2_. Macromol. Mater. Eng..

[61] Sipos L. (2010). A Process for Preparing a Polymer having a 2,5-Furandicarboxylate Moiety within the Polymer Backbone and Such (Co)Polymers, (Furanix Technologies B.V.). W.O. Patent.

[62] Knoop R.J.I., Vogelzang W., Van Haveren J., Van Es D.S. (2013). High molecular weight poly(ethylene-2,5-furanoate); critical aspects in synthesis and mechanical property determination. J. Polym. Sci. Part A.

[63] Hong S., Min K.-D., Nam B.-U., Park O.O. (2016). High molecular weight bio furan-based co-polyesters for food packaging applications: Synthesis, characterization and solid-state polymerization. Green Chem..

[64] Duh B. (2001). Reaction Kinetics for Solid-State Polymerization of Poly(ethylene terephthalate). J. Appl. Polym. Sci..

[65] Weissmann D., Kutz M. (2011). PET Use in Blow Molded Rigid Packaging. Applied Plastics Engineering Handbook.

[66] Gupta V.B., Bashir Z., Fakirov S. (2002). PET Fibers, Films, and Bottles. Handbook of Thermoplastic Polyesters: Homopolymers, Copolymers, Blends and Composites.

[67] Ros-Chumillas M., Belissario Y., Iguaz A., López A. (2007). Quality and shelf life of orange juice aseptically packaged in PET bottles. J. Food Eng..

[68] Berkowitz S. (1984). Viscosity–molecular weight relationships for poly(ethylene terephthalate) in hexafluorois opropanol–pentafluorophenol using SEC–LALLS. J. Appl. Polym. Sci..

[69] Karayannidis G.P., Kokkalas D.E., Bikiaris D.N. (1993). Solid-state polycondensation of poly(ethylene terephthalate) recycled from postconsumer soft-drink bottles. I. J. Appl. Polym. Sci..

[70] Pohl H.A. (1954). Determination of carboxyl end groups in a polyester, polyethylene terephthalate. Anal. Chem..

[71] Ravindranath K., Mashelkar R.A. (1981). Modeling of Poly(ethylene Terephthalate) Reactors. I. A Semibatch Ester Interchange Reactor. J. Appl. Polym. Sci..

[72] Mallon F.K., Ray W.H. (1998). Modeling of solid-state polycondensation. II. Reactor design issues. J. Appl. Polym. Sci..

[73] Ma Y., Agarwal U.S., Sikkema D.J., Lemstra P.J. (2003). Solid-state polymerization of PET: Influence of nitrogen sweep and high vacuum. Polymer.

[74] Ma Y., Agarwal U.S. (2005). Solvent assisted post-polymerization of PET. Polymer.

[75] Bikiaris D.N., Achilias D.S., Giliopoulos D.J., Karayannidis G.P. (2006). Effect of activated carbon black nanoparticles on solid state polymerization of poly(ethylene terephthalate). Eur. Polym. J..

[76] Achilias D.S., Karandrea E., Triantafyllidis K.S., Ladavos A., Bikiaris D.N. (2015). Effect of organoclays type on solid-state polymerization (SSP) of poly(ethylene terephthalate): Experimental and modelling. Eur. Polym. J..

[77] Achilias D.S., Gerakis K., Giliopoulos D.J., Triantafyllidis K.S., Bikiaris D.N. (2016). Effect of high surface area mesoporous silica fillers (MCF and SBA-15) on solid state polymerization of PET. Eur. Polym. J..

[78] Kaushik A., Gupta S.K. (1992). A molecular model for solid-state polymerization of nylon 6. J. Appl. Polym. Sci..

[79] Kulkarni M.R., Gupta S.K. (1994). Molecular Model for Solid-state Polymerization of Nylon 6. II. An Improved Model. J. Appl. Polym. Sci..

[80] Gaymans R.J., Amirtharaj J., Kamp H. (1982). Nylon 6 Polymerization in the Solid State. J. Appl. Polym. Sci..

[81] Li L.F., Huang N.X., Tang Z.L., Hagen R. (2001). Reaction kinetics and simulation for the solid-state polycondensation of nylon 6. Macromol. Theory Simul..

[82] Duh B. (2002). Semiempirical rate equation for solid state polymerization of poly(ethylene terephthalate). J. Appl. Polym. Sci..

[83] Wu D., Chen F., Li R., Shi Y. (1997). Reaction kinetics and simulations for solid-state polymerization of poly(ethylene terephthalate). Macromolecules.

