# Supplementary material for: Solid-State Polymerization of Poly(Ethylene Furanoate) Biobased Polyester, II: An Efficient and Facile Method to Synthesize High Molecular Weight Polyester Appropriate for Food Packaging Applications
Source: Polymers (Basel). 2018 Apr 25;10(5):471. doi: 10.3390/polym10050471 (PMC6415450; doi:10.3390/polym10050471)
Supplement: Supplementary file 1 [file polymers-10-00471-s001.pdf]

polymers

MDPI

Supplementary

Solid-State Polymerization of Poly(Ethylene Furanoate) Biobased Polyester, II: An efficient and facile method to synthesize very High-Molecular-Weight polyester appropriate for food packaging applications

Nejib Kasmi [1], George Z. Papageorgiou [2,*], Dimitris S. Achilias [1] and Dimitrios N. Bikiaris [1,*]

[1] Laboratory of Polymer Chemistry and Technology, Department of Chemistry, Aristotle University of Thessaloniki, GR-541 24, Thessaloniki, Macedonia, Greece; nejibkasmi@gmail.com (N.K.); axilias@chem.auth.gr (D.S.A.)

[2] Chemistry Department, University of Ioannina, P.O. Box 1186, 45110 Ioannina, Greece

* Correspondence: dbic@chem.auth.gr (D.N.B.); gzpap@cc.uoi.gr (G.Z.P.); Tel.: +30-231-0997812 (D.N.B.); +30-265-1008354 (G.Z.P.)

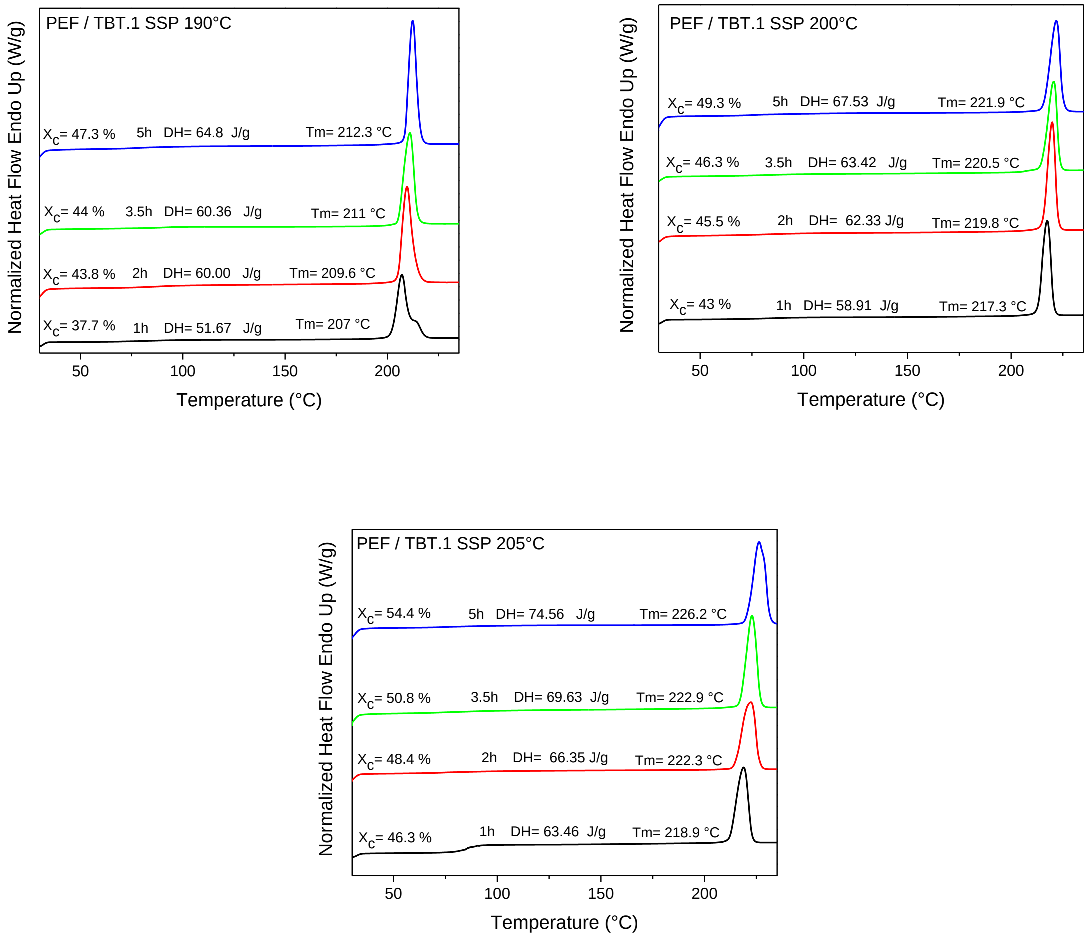

Figure S1. DSC thermograms of PEF/TBT.1 samples prepared after SSP at different temperatures and times.

polymers

MDPI

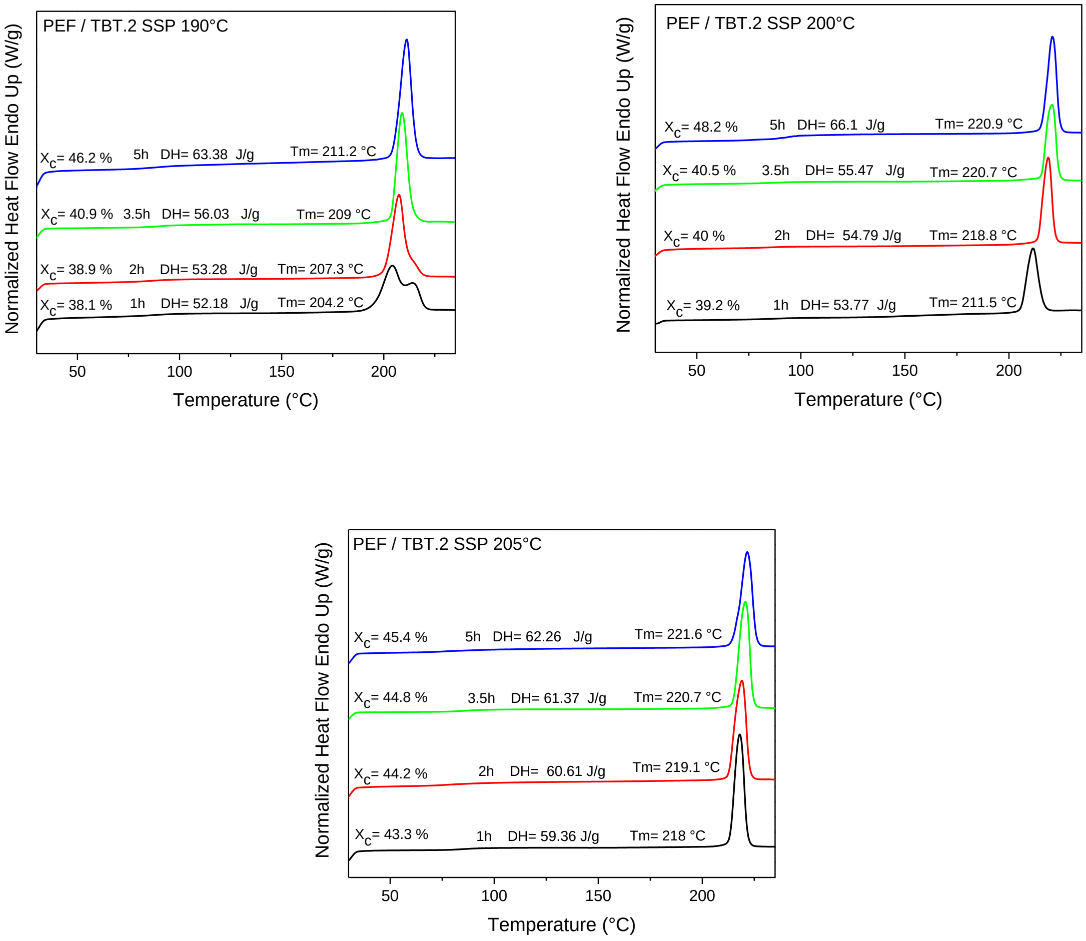

Figure S2. DSC thermograms of PEF/TBT.2 samples prepared after SSP at different temperatures and times.
